# Assessment of extended-spectrum β-lactamases and integrons among *Enterobacteriaceae* in device-associated infections: multicenter study in north of Iran

**DOI:** 10.1186/s13756-016-0143-2

**Published:** 2016-12-01

**Authors:** Masoumeh Bagheri-Nesami, Alireza Rafiei, Gohar Eslami, Fatemeh Ahangarkani, Mohammad Sadegh Rezai, Attieh Nikkhah, Azin Hajalibeig

**Affiliations:** 1 Infection Diseases Research Center with Focus on Nosocomial Infection, Mazandaran University of Medical Sciences, Sari, Iran; 2 Molecular and Cell Biology Research Center, Department of Immunology, Faculty of Medicine, Mazandaran University of Medical Sciences, Sari, Iran; 3 Department of Clinical Pharmacy, Faculty of Pharmacy, Mazandaran University of Medical Sciences, Sari, Iran; 4 Student Research Committee, Antimicrobial Resistance Research Center, Mazandaran University of Medical Sciences, Sari, Iran; 5 Traditional and Complementary Medicine Research Center, Mazandaran University of Medical Sciences, Sari, Iran

## Abstract

**Background:**

Device-associated nosocomial infections (DA-NIs), due to MDR *Enterobacteriaceae*, are a major threat to patient safety in ICUs. We investigated on Extended-spectrum β-lactamases (ESBL) producing *Enterobacteriaceae* and incidence of integrons in these bacteria isolated from ventilator-associated pneumonia (VAP) and catheter-associated urinary tract infections (CAUTIs) in 18 governmental hospitals in the north of Iran.

**Methods:**

In this cross-section study, the antibiotic susceptibility test was performed using the MIC method; also, phenotypically detection of ESBL-producing bacteria was carried out by the double-disk synergy (DDS) test. Presence of ESBL-related genes and integron Classes 1 and 2 was evaluated by the PCR method.

**Results:**

Out of a total of 205 patients with DA-NIs, *Enterobacteriaceae* were responsible for (72.68%) of infections. The most common DA-NIs caused by *Enterobacteriaceae* were VAP (77.18%), CAUTI (19.46%), and sepsis due to VAP (3.35%). The most frequently *Enterobacteriaceae* were; *Klebsiella pneumoniae* 75 (24; 32% ESBL positive), *E. coli* 69 (6; 8.69% ESBL positive) and *Enterobacter spp*. 5 (5; 100% ESBL positive). Distribution of ESBL-related genes was as follows: bla-SHV (94.3%), bla-CTX (48.6%), bla-VEB (22.9%) and bla-GES (17.14%). The incidence rate of integron class 1 and class 2 was (82.92%) and (2.9%) respectively. Eight types of ESBL-producing bacteria were observed.

**Conclusions:**

Due to the fact that the emergence rate of ESBL *Enterobacteriaceae* is increasing in DA-NIs, co-incidence of different types of ESBL genes with integrons in 75–100% of strains in our study is alarming for clinicians and healthcare safety managers. Therefore, regional and local molecular level estimations of ESBLs that are agents of DA-NIs are critical for better management of empiric therapy, especially for patients in ICUs.

**Electronic supplementary material:**

The online version of this article (doi:10.1186/s13756-016-0143-2) contains supplementary material, which is available to authorized users.

## Background

Device-associated nosocomial infections (DA-NIs), especially ventilator-associated pneumonia (VAP) and catheter-associated urinary tract infections (CAUTIs) pose the greatest threat to patient safety in the ICUs [1–3]. VAP is the most lethal among the two, however, CAUTIs are the most common *Enterobacteriaceae* that have been indicated as the most common cause of extended-spectrum β-lactamases (ESBL) producing bacteria in ICUs. These bacteria have a plethora of resistance mechanisms and often use multiple mechanisms against the same antibiotic or use a single mechanism to affect multiple antibiotics. Resistance to broad-spectrum cephalosporin is spreading quickly among *Enterobacteriaceae* and this is mostly related to acquisition of ESBL genes. Isolates that express ESBL phenotypes and hydrolize the beta lactam antibiotics are often multiple drug resistant (MDR) [4, 5]. The commonly genes related to the ESBL phenotype are sulf-hydryl variable (SHV), cefotaxime-beta lactamases (CTX), Vietnam extended-spectrum β-lactamase (VEB) and Guyana Extended-Spectrum ß-lactamases (GES) genes. Integrons as mobile DNA elements, are capable of detention and excision of antibiotic-resistant genes. Integrons achieve this by site-specific recombination. The different combinations of gene cassettes can contribute to the diverse genetic organization of integrons. There are five different classes of integrons. Class 1 integrons are the most common type that are present in *Enterobacteriaceae*.

Class 2 integrons are associated with the Tn7 transposon, whose transposition activity is directed at specific attachment sites on chromosomes or plasmids. Many of the antibiotic-resistant genes found in clinical isolates of *Enterobacteriaceae* are part of a gene cassette inserted into an integron [6]. Due to the potential of integrons to capture and collect gene cassettes, it is likely that incidence of MDR bacteria such as ESBL-producing *Enterobacteriaceae*, will become more prevalent in the future and integrons will continue to threaten the usefulness of antibiotics as therapeutic agents [6–8]. ESBL genes can be located on integrons, which may facilitate the spread of such genetic elements. To the best of our knowledge, this study is the first of its kind on ESBL-producing *Enterobacteriaceae* and incidence of integrons in these bacteria isolated from VAP and CAUTI as a major threat to patient safety in ICU wards, which was conducted in 18 governmental hospitals of Mazandaran province (The largest province in the north of Iran in terms of area and population).

## Methods

### Study population and DA-NIs definitions

This cross-sectional study was conducted in 18 governmental hospitals that overall contained 1200 ward beds and 100 intensive care unit beds, in Mazandaran province, located in the north of Iran, during 2014 and 2015. This study was approved by the Ethics Committee of Mazandaran University of Medical Sciences (Code No: 879 Date: July 9, 2014).

DA-NIs were defined as: Catheter-Associated Urinary Tract Infection; patient with a urinary catheter that had fever, dysuria, frequency, flank pain, suprapubic pain, nausea and vomiting. In addition, the urine culture was positive for 10^5^ colony forming units per mL or more, with no more than two microorganisms isolated or, must have had at least two symptoms such as fever, dysuria, frequency, flank pain, suprapubic pain, nausea and vomiting plus pyuria.

Ventilator-Associated Pneumonia; Ventilator-associated pneumonia was indicated in a mechanically ventilated patient with a chest radiograph that showed new or progressive infiltrates, cavitation, consolidation, or pleural effusion 48 h after hospitalization. The patient must have had at least one of the following criteria: new onset of purulent sputum or change in character of sputum; organism cultured from blood or from a specimen obtained by tracheal aspirate, bronchoalveolar lavage or bronchial brushing, or biopsy.

Sepsis due to VAP; in patients ventilated more than 72 h, and bacteria separated from positive blood culture and tracheal tube aspirate positive culture were similar; while the patients had the symptoms of systemic inflammatory response syndrome (SIRS).

For all the patients whom were subject to ventilator and urinary catheter, certain prevention strategies were used against VAP and CAUTIs.

The prevention strategies for CAUTIs: insert catheters just for appropriate indications; leave catheters only as long as needed; only trained nurses insert and maintain catheters; hand hygiene; Insert catheters using aseptic technique and sterile equipment; aseptic insertion, maintain a closed drainage system; maintain unobstructed urine flow.

The prevention strategies for VAP are: elevation of the head of the bed; oral hygiene care; Prophylaxis interventions for peptic ulcer disease and deep vein thrombosis.

### Sampling and microbiological methods

For VAP, a deep tracheal aspirate from the endotracheal tube was obtained, and for CAUTI, urine was aseptically aspirated from the sampling port of the urinary catheter for performing gram stain and culture on selective media. Sampling was done by the head nurses and the samples were immediately transported in a transport medium to the microbiology laboratory. All the samples were routinely cultured on MacConkey and blood agar plates. Blood samples were cultured in Blood culture bottles. Isolates were identified at the species level using standard biochemical tests and microbiological methods [9, 10].

### Antibiotic susceptibility test

Susceptibility of the clinical isolates to routinely used antibiotics was determined by the standard broth dilution (micro dilution) technique. MIC was determined according to the recommendations of the standard protocol of CLSI 2010. The antibiotics were purchased from Sigma chemical company. Antibiotics used in this study were Amikacin, Ciprofloxacin, Imipenem, Gentamicin, Ceftazidime, Tobramycin, Piperacillin-Tazobactam, Cefepime, Colistin and Co-trimoxazole.

### Phenotype detection of extended-spectrum beta-lactamase (ESBL) producing *enterobacteriaceae*

ESBL-producing *Enterobacteriaceae* was detected using the double-disk synergy (DDS) test [11, 12]. ESBL’s presence was assayed using the following antibiotic disks (MAST, UK): cefotaxime (30 μg), cefotaxime/clavulanic acid (30/10 μg), ceftazidime (30 μg), and ceftazidime/clavulanic acid (30/10 μg). *Escherichia coli* ATCC 25922 strain served as positive controls.

### DNA extraction and detection of ESBL-related genes

*Enterobacteriaceae* that were phenotypically confirmed as ESBL, were evaluated for ESBL-related genes. DNA of ESBL-positive *Enterobacteriaceae* was extracted using a commercial gene extraction kit (DNA Zist, Iran) according to the company’s instructions. ESBL-positive strains were screened by the PCR method for genes bla CTX, bla VEB, bla GES, bla SHV and also integrons class 1 and class 2. The set of primers and PCR amplification conditions are available in Additional file 1. After performing the PCR reaction, electrophoresis of PCR products was carried out in 2% agarose gel at 70 voltage for 50 min. Then, results were evaluated under UV light on the UV Trans illuminator. In all the experiments, the following reference strains were used as positive controls: *K. pneumoniae* 7881 (*CTXM*), *K. pneumoniae* 7881 strain (containing *SHV*), *P. aeruginosa* ATCC 27853 (VEB-1), and *K. pneumoniae* (GES). *E. coli* 96 K062 was used as a positive control for classes 1 and 2 integrons. A non-ESBL-producing strain (*E. coli* ATCC 25922) was used as negative control.

### Statistical analysis

Data were analyzed using SPSS software version 16. Descriptive statistics, Chi- square and Fisher’s exact tests were used for statistical analysis.

## Results

Out of total of 205 hospitalized patients with DA-NIs in ICU wards of the mentioned hospitals during 2014–2015, *Enterobacteriaceae* were responsible of 149 (72.68%) of DA-NIs. The most frequently found *Enterobacteriaceae* were; *Klebsiella pneumoniae* 75 (24; 32% ESBL positive & 51; 68% ESBL negative), *E. coli* 69 (6; 8.69% ESBL positive & 63; 91.30% ESBL negative) and *Enterobacter* spp. 5 (5; 100% ESBL positive). The most common DA-NIs caused by *Enterobacteriaceae* were VAP (77.18%), CAUTI (19.46%) and sepsis due to VAP (3.35%).

The demographic feature of patients with DA-NIs caused by ESBL *Enterobacteriaceae* was as follows; 27 VAP patients (15; 55.5% male and 12; 44.4% female) with average age of 66.5 ± 20.17 years and average duration of hospitalization in the ICU of 28.37 ± 20.03 days; three CAUTI patients (1; 33.3% male and 2; 66.6% female) with average age of 45.66 ± 21.93 years and average duration of hospitalization in the ICU of 19.33 ± 15.63 days; five patients of sepsis due to VAP (2;40% male and 3;60% female) with average age of 60.25 ± 14.79 years and average duration of hospitalization in the ICU of 10.75 ± 1.89 days.

**Fig. 1:**
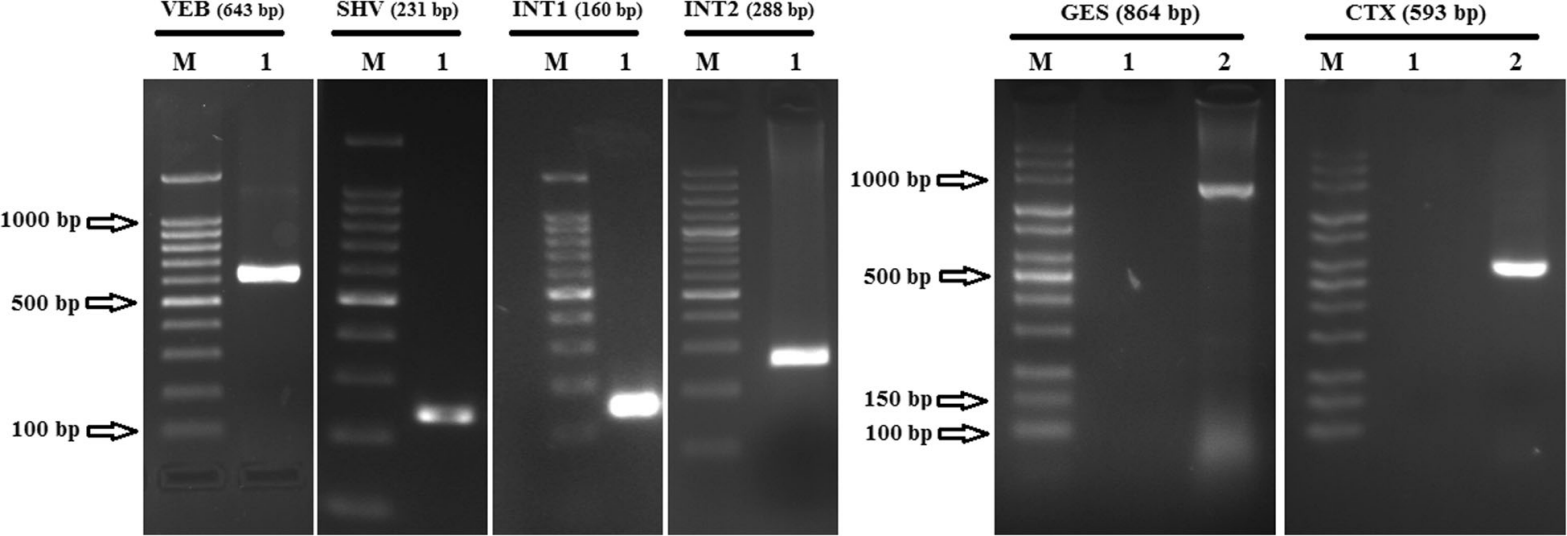
Agarose gel showing the strains containing, VEB, SHV, int1 (integron class1), int2 (integron class 2), GES and CTX genes

In total, the distribution of ESBL-related genes was 33 (94.3%) bla-SHV, 17 (48.6%) bla-CTX, 8 (22.9%) bla-VEB and 6 (17.14%) bla-GES. Figure 1 that is the illustration of agarose gel, shows the strains containing VEB, SHV, int1 (integron class1), int2 (integron class 2), GES and CTX genes. The Antibiotic susceptibility pattern of *Enterobacteriaceae* containing ESBL-related genes is shown in detail in Table 1. The rate of antibiotic resistance among strains containing SHV gene was 27.3–78.8% whereas the rate of sensitivity was 6.1–48.5%. On the other hand, Gentamicin and Imipenem had the highest resistance and sensitivity rates respectively. The rate of antibiotic resistance among strains containing CTX gene was 41.2–88.2%, whereas the rate of sensitivity was 5.9–35%. In addition, Gentamicin and Ciprofloxacin had the highest resistance and sensitivity rates respectively. The rate of antibiotic resistance among strains containing VEB gene was 12.5–87%, whereas the rate of sensitivity was 12.5–75% and Ceftazidime and Ciprofloxacin had the highest resistance and sensitivity rates respectively. The rate of antibiotic resistance among strains containing GES gene was 16.7–83.3% whereas the rate of sensitivity was 16.7–66.7%. On the other hand, Cefepime and Imipenem had the highest resistance and sensitivity rates respectively. The incidence of integrons class 1 and class 2 was 29 (82.92%) and 1 (2.9%) respectively. Antibiotic susceptibility pattern of integron-positive *Enterobacteriaceae* is shown in Table 2.

**Table 1:** Antibiotic susceptibility pattern of *Enterobacteriaceae* containing ESBL related genes

		*Klebsiella pneumoniae*				*Enterobacter .spp*				*E.coli*			
		*N* = 24				*N* = 5				*N* = 6			
		Positive ESBL related genes											
		SHV	CTX	VEB	GES	SHV	CTX	VEB	GES	SHV	CTX	VEB	GES
		*N* = 22	*N* = 14	*N* = 4	*N* = 4	*N* = 5	*N* = 2	*N* = 3	*N* = 1	*N* = 6	*N* = 1	*N* = 1	*N* = 1
Amikacin	R	50	50	25	25	60	100	33	0	66.6	100	100	100
	I	27.2	28.5	50	25	40	0	66	100	16.6	0	0	0
	S	22.7	21.5	25	50	0	0	0	0	16.6	0	0	0
Ciprofloxacin	R	40.9	50	0	25	0	0	0	0	33.3	0	0	100
	I	31.8	28.5	50	50	0	0	0	0	-	-	-	-
	S	27.2	21.5	50	25	100	100	100	100	66.6	100	100	0
Imipenem	R	36.3	50	25	50	0	0	0	0	16.6	0	0	0
	I	27.2	28.5	25	25	20	50	33	100	16.6	0	0	100
	S	36.3	21.5	50	25	80	50	66	0	66.6	100	100	0
Gentamicin	R	81.8	85.7	50	100	80	100	33	0	83.33	100	100	100
	I	4.54	0	0	0	20	0	33	100	16.6	0	0	0
	S	13.63	14.2	50	0	0	0	33	0	-	-	-	-
Ceftazidime	R	72.7	57.14	75	50	60	50	100	100	50	100	100	0
	I	27.2	35.7	25	25	40	50	0	0	33.3	0	0	100
	S	13.63	7.1	0	25	0	0	0	0	16.6	0	0	0
Tobramycin	R	59	64.28	50	25	60	100	33	0	33.3	0	0	100
	I	31.8	28.57	50	50	0	0	0	0	16.6	0	100	0
	S	9.1	7.1	0	25	40	0	66	100	50	100	0	0
Piperacillin-Tazobactam	R	63.6	64.28	50	75	60	0	33	100	16.6	0	0	0
	I	31.8	28.57	25	25	0	50	66	0	66.6	100	100	100
	S	4.54	7.1	25	0	40	50	0	0	16.6	0	0	0
Cefepime	R	72.7	85.71	25	100	60	100	66	100	33.3	0	100	0
	I	9.1	7.1	25	0	20	0	0	0	16.6	0	0	100
	S	18.18	7.1	50	0	20	0	33	0	50	100	0	0
Colistin	R	72.7	71.42	50	25	40	100	33	0	33.3	0	0	100
	I	27.2	14.2	25	25	20	0	0	0	16.6	100	0	0
	S	13.63	14.2	25	50	40	0	66	100	50	0	100	0
Co-trimoxazole	R	81.8	85.71	75	75	80	100	66	0	33.3	0	100	100
	I	-	-	-	-	0	0	0	0	-	-	-	-
	S	18.2	14.2	25	25	20	0	33	100	66.6	100	0	0

*R* resistant, *I* intermediate, *S* sensitive

**Table 2:** Antibiotic susceptibility pattern of integron positive *Enterobacteriaceae*

		*Klebsiella pneumoniae*		*Enterobacter .spp*		*E.coli*	
		*N* = 24		*N* = 5		*N* = 6	
		Integron class 1	Integron class 2	Integron class 1	Integron class 2	Integron class 1	Integron class 2
		*N* = 20	*N* = 1	*N* = 4	*N* = 0	*N* = 6	*N* = 0
Amikacin	R	55	100	75	-	66.6	-
	I	25	0	25		16.6	
	S	20	0	0		16.6	
Ciprofloxacin	R	45	100	0	-	33.3	-
	I	35	0	0		-	
	S	20	0	100		66.6	
Imipenem	R	35	0	0	-	16.6	-
	I	30	0	25		16.6	
	S	35	100	75		66.6	
Gentamicin	R	80	100	75	-	83.3	-
	I	5	0	0		16.6	
	S	15	0	25		-	
Ceftazidime	R	55	100	50	-	50	-
	I	30	0	50		33.3	
	S	15	0	0		16.6	
Tobramycin	R	60	0	75	-	33.3	-
	I	30	100	0		16.6	
	S	10	0	25		50	
Piperacillin-Tazobactam	R	70	100	75	-	16.6	-
	I	30	0	25		66.6	
	S	0	0	0		16.6	
Cefepime	R	80	100	75	-	33.3	-
	I	10	0	25		16.6	
	S	10	0	0		50	
Colistin	R	60	0	50	-	33.3	-
	I	25	100	25		16.6	
	S	15	0	25		50	
Co-trimoxazole	R	85	100	75	-	33.3	-
	I	-	-	0		-	
	S	15	0	25		66.6	

*R* resistant, *I* intermediate, *S* sensitive

The rate of antibiotic resistance among integron class 1 positive strain was 35–85%. The only integron class 2 positive strain was *Klebsiella pneumoniae* and this isolate was resistant to all the antibiotics. Eight types of ESBL genes were seen among the isolates. Coincidence of each type of ESBL-producing bacteria and integron class 1 is shown in Table 3. Nine strains contained three ESBL genes (2 strains had GES, VEB, and SHV, 3 strains had GES, CTX, and SHV and 4 strains had VEB, CTX, and SHV). Fourteen strains contained 2 ESBL genes (10 strains had CTX and SHV, one strain had GES and SHV and three strains had VEB and SHV). Twelve strains had only one ESBL gene (11 strains contained SHV and one strain had CTX). Coincidence of isolates that contained different types of ESBL genes and integron class 1 was 75–100%, which was statistically significant (*P* > .05).

**Table 3:** Coincidence of ESBL genes types and integron class 1 among *Enterobacteriaceae* isolated

ESBL types	Number	Coincidence with integron class 1	*P* value
GES, VEB, SHV	2 (5.71)	2 (100)	0.00
GES, CTX, SHV	3 (8.57)	3 (100)	0.00
VEB, CTX, SHV	4 (11.42)	3 (75)	0.00
CTX, SHV	10 (28.57)	8 (80)	0.01
GES, SHV	1 (2.85)	1 (100)	0.00
VEB, SHV	3 (8.57)	1 (100)	0.00
SHV	11 (31.42)	11 (100)	0.02
CTX	1 (2.85)	1 (100)	0.00

## Discussions

DA-NIs due to MDR bacteria are a serious threat to patient safety, being among of the most serious causes of morbidity, mortality and economic burden in developing countries such as Iran. Various studies have shown that the DA-NIs are a serious issue in Iran [13–16] But no studies had specifically addressed and evaluated the ESBL genes and mobile genetic elements such as integrons in *Enterobacteriaceae* as common agents of DA-NIs patients in Iran. It was found in this study that ESBL-producing *Enterobacteriaceae* were causative agents of 23% of DA-NIs in the region. Rosenthal et al. surveyed DA-NIs in 55 ICUs of eight developing countries and found that VAP posed the greatest risk (41%), followed by CVC-related bloodstream infections (30%) and CAUTI (29%). On the other hand, they reported *Enterobacteriaceae* were agents of about 27% of VAPs and 42% of CAUTIs [16]. In our study, overall 56% of *Enterobacteriaceae* were resistant to the 3rd generation of cephalosporin, and imipenem was the most effective antibiotic. Similar to the results of this study, Rosenthal et al. reported that 51% of *Enterobacteriaceae* isolates were resistant to ceftriaxone [16]. Salomao et al. reported that in five ICUs in three urban hospitals of Brazil during a three-year period, VAP rate was 20.9 per 1000 ventilator days, CAUTI rate was 9.6 per 1000 catheter days, and *Enterobacteriaceae* were agents of 22.8% of DA-NIs. *Enterobacteriaceae* in their study were resistant to ceftriaxone in 96.7% of the cases and resistance to ceftazidime was seen in 79.3% of the cases [17]. Guanche-Garcell in Cuba determined the incidence rate of DA-NIs to be 17.0% for VAP, 4.4% for CAUTI and 1% for CVC. They found that overall 51.7% of all DA-NIs were caused by *Enterobacteriaceae*. *Escherichia coli* was responsible for VAP and CAUTI for 15.4 and 53.8% of the cases respectively. VAP and CAUTI due to *Klebsiella* spp. were 23.1 and 15.4% respectively. The rate of VAP caused by *Enterobacteriaceae* in the mentioned studies was lower than the findings of this study.

ESBL *Enterobacteriaceae* pose unique challenges to infection control professionals and antibacterial-discovery scientists [18, 19]. In this study, prevalence of ESBL-related genes was; 94.3% for SHV, 48.6% for CTX, 22.9%for VEB and 17.14% for GES. β-Lactams not only are extensively used for treatment of common infections, but also frequently used as prophylaxis before surgery. *Enterobacteriaceae* are of clinical importance since they cause infections, especially in patients that use the devices in ICUs. ESBL-producing *Enterobacteriaceae* are often MDR, further limiting the therapeutic options. In the present study, Co-resistance with fluoroquinolones, aminoglycosides, trimethoprim, and cephalosporins were found in average in 33–80% of ESBL bacteria. Knowledge of the local epidemiology of ESBL DA-NIs in molecular level is very important. For an immunocompromised patient, such as patients admitted in ICUs with DA-NIs caused by ESBL-producing *Enterobacteriaceae*, administration of an ineffective antibiotic can be lethal. The rate of ESBL varies geographically but it is increasing fast in the region; for example in our previous study on ESBL-Escherichia coli uropathogens of pediatrics in 2014, the rate of ESBL genes were SHV (44%), CTX (28%), VEB (8%), and GES (0%); but in this study, the rate of these genes were 16.6–100% among *E. coli* [20]. In addition, Khorshidi et al. in Kashan and Khosravi et al. in Ahvaz, reported the rates of bla-SHV to be about 50%, which is lower than the findings of this study [21, 22]. The prevalence of ESBL-producing *Enterobacteriaceae* in Iran has been reported in different rates by phenotypic confirmatory test.

For example Behroozi et al., reported that 21% of *E. coli* and 12% of *K. pneumonia* isolates were ESBL producers; on the other hand, Feizabadi et al. reported that 72% of *K. pneumonia* strains isolated from Tehran hospitals were ESBL producers. Also in our previous study on bacteria isolated from patients with chronic sinusitis, the rate of ESBL-producing bacteria was 28.75–37.03% among *Enterobacteriaceae* [12, 23, 24]. High prevalence of ESBL-producing *Enterobacteriaceae* that are agents of DA-NIS in ICUs, represent the rising problem of antibiotic resistance rate in the ICUs, which is caused by suboptimal infection control in hospitals and new mutations of resistant genes. Transmission of the genes of ESBL enzymes can occur by horizontal gene transfer. Integrons, as common genetic mobile elements, are associated with ESBL genes. In this study, the presence of class 1 integron varied from 80 to 100% among ESBL-producing species; also eight types of ESBL-producing *Enterobacteriaceae* were found. All these types significantly correlate with the incidence of integron class 1 (*p* ≥ 0.02). Similar to these results, association of certain beta-lactamase genes with class 1 integrons by location of bla genes within integron platforms (blaVIM, blaIMP, blaGES, blaVEB, blaCTX-M-2/-9, and blaCMY) or by sharing the same plasmid context (blaTEM and blaSHV) among *Enterobacteriaceae*, has been previously reported in several studies [25–28].

## Conclusions

The emergence of ESBL *Enterobacteriaceae* among DA-NIs is increasing in the Mazandaran province. The emergence of coincidence of different types of ESBL genes with integrons in 75–100% of strains is really dangerous and alarming for clinicians and healthcare safety managers. Due to the fact that prevalence of ESBL-producing strains can vary greatly from one ward to another, and even for a given ward in different points in time, therefore estimating regional and local ESBL agents of DA-NIs in molecular level in high-risk wards, at least once a year, could be useful in clinical decision-making of empiric therapy, especially for patients in ICUs.

## Additional file

Additional file 1: Table S1. The set of primers and PCR amplification conditions. (DOCX 12 kb)

## Abbreviations

CAUTI: Catheter-associated urinary tract infections
CTX: Cefotaxime-beta lactamases
CVC: Central venous catheter
DA-NIs: Device associated nosocomial infections
ESBL: Extended-spectrum beta-lactamases
GES: Guyana Extended-Spectrum ß-lactamases
ICU: Intensive care unit
MIC: Minimum inhibitory concentration
NIs: Nosocomial infection
PCR: Polymerase chain reaction
SHV: Sulf-hydryl variable
VAP: Ventilator-associated pneumonia
VEB: Vietnam extended-spectrum β-lactamase
