# Supplementary material for: Assessment of extended-spectrum β-lactamases and integrons among Enterobacteriaceae in device-associated infections: multicenter study in north of Iran
Source: Antimicrob Resist Infect Control. 2016 Dec 1;5:52. doi: 10.1186/s13756-016-0143-2 (PMC5134273; doi:10.1186/s13756-016-0143-2)
Supplement: Additional file 1: Table S1. — The set of primers and PCR amplification conditions. (DOCX 12 kb) [file 13756_2016_143_MOESM1_ESM.docx]

Additional file 1: Table S1. The set of primers and PCR amplification conditions

| **PCR product**  **size** | **Thermal cycling condition** | **Primer used(5'-3')** | **Target ‎genes** |
| --- | --- | --- | --- |
| 593 bp | 94°c 5min→40×[ 94°c 45sec,  53.1°c 45sec, 72°c 1min] → 72°c 7 min | TTTGCGATGTGCAGTACCAGTAA  CGATATCGTTGGTGGCATA | **bla CTX** |
| 585 bp | 93°c 3min→40×[ 93°c 1min, 54.9°c 1min, 72°c 1min] → 72°c 7 min | CGACTTCCATTTCCCGATGC GGACTCTGCAACAAATACGC | **bla VEB** |
| 846 bp | 1 cycle of 5 min at 95°C; 30 cycles of 1 min at 95°C, 45 sec at 55°C, 30 sec at 72°C; 1 cycle of 8 min at 72°C | ATGCGCTTCATTCACGCAC CTATTTGTCCGTGCTCAGG | **bla GES** |
| 231 bp | 1 cycle of 3 min at 94°C; 35 cycles of 30 sec at 94°C, 1 min at 60°C, 1 min at 72°C; 1 cycle of 7 min at 72°C | AAGATCCACTATCGCCAGCAG  ATTCAGTTCCGTTTCCCAGCGG | **bla SHV** |
| 160 bp | 1 cycle of 2 min at 94°C; 35 cycles of 30 sec at 94°C, 30 sec at 55°C, 30 sec at 72°C; 1 cycle of 3 min at 72°C | CAGTGGACATAAGCCTGTTC  CCCGAGGCATAGACTGTA | **INT1** |
| 288 bp | 1 cycle of 5min at 95°C; 30 cycles of 45 sec at 94°C, 40 sec at 58°C, 1 min at 72°C; 1 cycle of 7 min at 72°C | TTGCGAGTATCCATAACCTG  TTACCTGCACTGGATTAAGC | **INT2** |
